# State-of-the-Art Review: Transjugular Tricuspid Valve Replacement Using the LuX-Valve Plus System

**DOI:** 10.1016/j.shj.2026.100792

**Published:** 2026-01-06

**Authors:** Erwan Donal, Augustin Coisne, Julien Ternacle, Guillaume L’Official, Julien Dreyfus, Mohammed Nejjari, Lionel Leroux, Ole de Backer, Azeem Latib, Juan F. Granada, Ralph Stephan von Bardeleben, Thomas Modine

**Affiliations:** a University of Rennes, CHU Rennes, Inserm, Rennes, France; b University of Lille, Inserm, CHU Lille, Institut Pasteur de Lille, Lille, France; c Cardiovascular Research Foundation, New York, New York, USA; d Unité Médico-Chirurgicale des Valvulopathies Hôpital Cardiologique Haut-Lévêque, CHU de Bordeaux, Pessac, France; e Centre de Recherche Cardio-Thoracique de Bordeaux, University of Bordeaux, INSERM, Pessac, France; f Cardiology Department, Centre Cardiologique du Nord, Paris, France; g The Heart Center, Rigshospitalet, Copenhagen University Hospital, Copenhagen, Denmark; h Institute for Klinisk Medicine, University of Copenhagen, Copenhagen, Denmark; i Montefiore-Einstein Center for Heart and Vascular Care, Montefiore Medical Center, Albert Einstein College, Bronx, New York, USA; j Department of Cardiology, Universitätsmedizin Mainz of the Johannes Gutenberg-University of Mainz, Mainz, Germany; k Structural Heart Valve Center, Institut de Cardiologie de Montréal, Montreal Heart Institute, Montréal, Canada

**Keywords:** Imaging guiding, Transcatheter tricuspid valve replacement, Tricuspid regurgitation

## Abstract

Severe tricuspid regurgitation remains a major therapeutic challenge, particularly in patients with extensive annular dilation or advanced right heart remodeling. Transcatheter tricuspid valve replacement(TTVR) has emerged as a promising alternative for anatomically complex and high‑risk patients who are not suitable for surgery or transcatheter edge‑to‑edge repair. Among currently available systems, the LuX‑Valve Plus device offers a unique transjugular approach and anchoring strategy. This state‑of‑the‑art review provides a comprehensive overview of the LuX‑Valve Plus system, including technical characteristics, procedural steps, multimodality imaging guidance, and key anatomical considerations for patient selection. Drawing on current literature and expert experience, the review describes a step‑by‑step procedural framework with a focus on anatomical screening, computed tomography–based planning, intraprocedural three‑dimensional transesophageal echocardiography, and the use of advanced imaging modalities such as fusion imaging. Practical tips are provided to optimize device positioning, septal anchoring, and leaflet interaction in the setting of highly variable anatomy. Overall, the LuX‑Valve Plus system broadens the spectrum of TTVR to anatomically challenging patients, and this review offers a practical guide for clinicians by offering detailed technical insights, imaging protocols, and procedural strategies essential for successful implantation and avoidance of complications.

## Highlight

• The Lux valve is a unique percutaneous prosthesis with a transjugular delivery approach and the ability to treat very large valve annuli.
• Optimal candidate selection requires high-resolution, multimodality imaging (gated cardiac computed tomography and transoesophageal echocardiography).
• Prospective multicenter studies are needed to validate the initial expectations.

## Graphical Abstract

Key features of the prosthesis.

**Figure:**
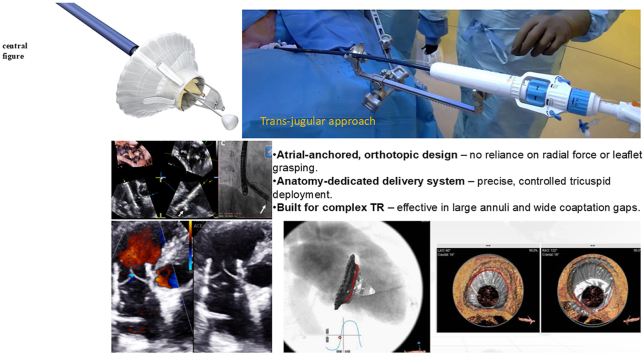

## Introduction

Severe tricuspid regurgitation (TR) is associated with significant morbidity and mortality, yet remains undertreated due to the high risk of surgical intervention in patients referred late and the anatomical limitations of transcatheter edge-to-edge repair (TEER).^1^^,^^2^ Recently, transcatheter tricuspid valve replacement (TTVR) has emerged as a viable therapeutic alternative in patients at high or prohibitive risk. Although the TRILUMINATE, Tri-Frand, and CLASP TR trials established transcatheter repair as a therapeutic option for TR, anatomical and clinical limitations have accelerated the development of TTVR.^3^, ^4^, ^5^ Among the available TTVR devices, the LuX-Valve Plus system offers a unique transjugular delivery approach and the ability to treat very large valve anatomy. This review provides a comprehensive overview of the LuX-Valve Plus device, from anatomical screening to intraprocedural guidance and early clinical outcomes, highlighting its role within the expanding TTVR landscape.^6^^,^^7^

## Device Overview

### Technical Characteristics of the LuX-Valve Plus System

**Figure 1:**
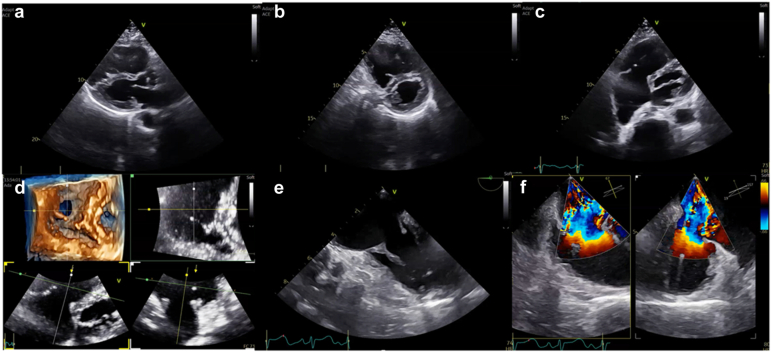
**TTE and TEE evaluation of a patient with torrential TR related to a carcinoid disease. (a and b)** Severe RV dilatation with diastolic septal shift of the interventricular septum. **(c and d)** Important tethering and restriction of the tricuspid leaflets and wide coaptation gap in 2D and 3D MPR. Significant thickening of the subvalvular apparatus was also observed in TTE **(e)** with torrential TR in color Doppler **(f)** Abbreviations: MPR, multiplanar reconstruction; RV, right ventricle; TEE, transesophageal echocardiography; TR, tricuspid regurgitation; TTE, transthoracic echocardiography.

**Figure 2:**
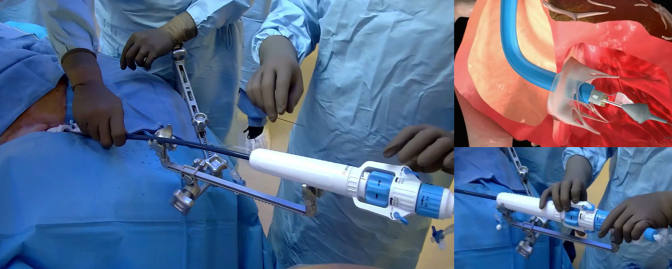
**Procedural room set-up and team positioning for transjugular tricuspid valve replacement procedures.**

The LuX-Valve Plus (Jenscare Biotechnology Co. Ltd, Ningbo, China) is an orthotopic TTVR system designed specifically for the unique anatomical and functional challenges of the tricuspid valve (Figure 1 and graphical abstract). Unlike transfemoral devices such as the EVOQUE system,^8^ the LuX-Valve Plus is delivered via a transjugular approach, providing a more coaxial trajectory to the tricuspid annulus and potentially reducing access-related tortuosity.^9^, ^10^, ^11^ Contrary to the common sense of cardiologists used to the femoral access, suggesting inherent limitations of transjugular access, our experience and the available data indicate that the right internal jugular (RIJ) approach is generally favorable for transcatheter tricuspid interventions. The RIJ provides a direct, coaxial trajectory to the tricuspid valve, facilitating system orientation, device steering, and controlled deployment. In practice, venous tortuosity is uncommon, anatomical constraints are usually limited, and access-site complications are rare when standard precautions are applied. Moreover, the dedicated delivery system is specifically designed to exploit this superior alignment, allowing smooth and intuitive manipulation. Although femoral access is more familiar in most catheterization laboratories, the jugular approach appears technically advantageous for tricuspid valve procedures and may offer improved procedural ergonomics and precision. Figure 2 illustrates procedural room setup and team positioning during transcatheter tricuspid valve replacement.

The system features a self-expanding nitinol frame with a trileaflet bovine pericardial valve and is available in a broad range of valve sizes (40 to 70 mm in 5 mm increments), making it particularly suited for patients with large tricuspid annuli, including those with torrential TR and advanced right heart remodeling. The device is anchored by a combination of anterior and posterior leaflet graspers and a unique interventricular septal anchor, providing secure fixation even in the absence of annular or leaflet calcification. These features enable atraumatic deployment while minimizing the risk of valve migration or paravalvular leak.

The delivery system is 33-F compatible and incorporates steerability to accommodate right atrial size and anatomic variation. Deployment is guided by a combination of fluoroscopy and transesophageal echocardiography (TEE), with precise positioning required to align the septal anchor and leaflet graspers before final release.

### Comparison With Other TTVR Systems

TTVR systems vary significantly in terms of delivery approach, anchoring strategy, valve sizing, and procedural complexity. Table 1 summarizes key technical differences among currently used systems.

**Table 1:** Key technical differences among currently TTVR systems

Device	Access route	Delivery system (French)	Anchoring mechanism	Valve sizes (mm)
LUX-VALVE PLUS	Transjugular	33 F	Septal anchor + anterior/posterior leaflet graspers	40, 45, 50, 55, 60, 65, 70
EVOQUE	Transfemoral	28 F	Intraannular skirt + leaflet anchors	44, 48, 52, 56 (selected sizes CE-approved)
CARDIOVALVE	Transfemoral	32 F	Leaflet graspers + atrial flange	M, L, XL
TRISOL	Transjugular	30 F	Axial force + leaflet anchors	1 size (40–53 mm)
TOPAZ	Transfemoral	29 F	Subannular anchors	45, 55
VDYNE	Transfemoral	28 F	RVOT anchor + proximal loop	5 sizes (42–56 mm)

Abbreviation: TTVR, transcatheter tricuspid valve replacement.

The LuX-Valve Plus stands out as the only widely studied system employing a transjugular approach, which offers a more direct line to the tricuspid valve and may be advantageous in patients with inferior vena cava offset or distorted venous anatomy. In addition, the broad valve size range (up to 70 mm) makes it uniquely capable of treating patients with massively dilated annuli, a population often ineligible for other devices. A notably low screening failure rate has been observed with the LuX-Valve Plus system, plausibly related to its limited reliance on precise leaflet morphology and comprehensive leaflet visualization, in contrast to leaflet-dependent transcatheter platforms.

Anchoring mechanisms differ substantially. While EVOQUE relies on leaflet engagement with an intra-annular skirt, the LuX-Valve Plus’ relies on multipoint anchoring, including a needle to anchor directly on the septum. It may provide greater stability in complex or heavily remodeled right hearts. However, the use of septal anchoring also necessitates precise imaging and procedural planning to avoid conduction system injury. Extreme interventricular septum–tricuspid annulus angulations identified on cardiac computed tomography (CT may adversely affect anchoring stability and paravalvular sealing. However, the transjugular approach offers improved coaxial alignment with the septum, potentially reducing the impact of unfavorable angulation on procedural feasibility.

In terms of early clinical experience, both systems have demonstrated excellent TR reduction, with >90% of patients achieving ≤1+ TR at 30 days.^6^^,^^12^ The LuX-Valve Plus' unique size availability and anchoring design support its use in anatomically extreme cases, whereas EVOQUE benefits from a simpler transfemoral delivery and growing commercial availability.

## Patient Selection and Anatomical Considerations

### Patient Selection and Clinical Context

The LuX-Valve Plus system has predominantly been used in a compassionate use setting, addressing a subset of patients with symptomatic severe or torrential TR who are ineligible for conventional surgery or TEER. This includes patients with excessive tricuspid annular dilation beyond the range of TEER systems, unfavorable leaflet morphology or large coaptation gaps, concomitant right heart failure or advanced organ dysfunction, and prior cardiac devices or procedures complicating leaflet access.

Patient eligibility is determined through heart team consensus, based on a comprehensive clinical, biological, imaging, anatomical, and hemodynamical assessment. The absence of formal exclusion criteria in early registries reflects a real-world cohort with high procedural complexity, often involving challenging right heart anatomy and multiple comorbidities.

### Anatomical Screening and Imaging Assessment

Optimal candidate selection for LuX-Valve Plus implantation requires high-resolution, multimodality imaging, tailored to address the unique anatomical variability of the tricuspid valve and right heart.

#### Echocardiography

**Figure 3:**
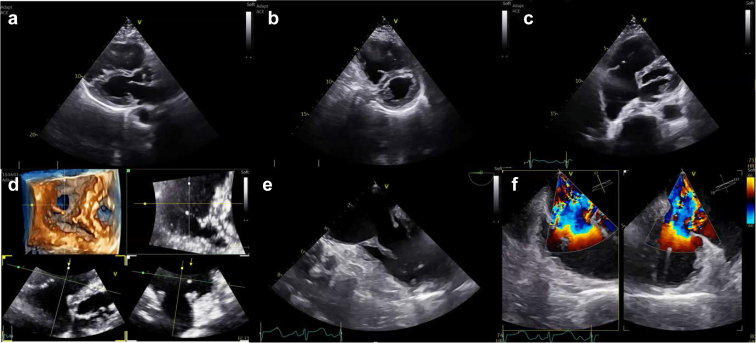
**Computed tomography analysis for TTVR screening using the Lux-Valve Plus system – Part 1. (a)** The annular area, perimeter, and maximum and minimum diameters are measured in diastole to determine the appropriate device size and percentage oversizing. **(b)** Dimensions of the fabric skirt plane are also measured. **(c and d)** The target positions of the device and septal anchor are modeled. **(e)** The distances between the center of the tricuspid annulus and the free wall of the right ventricle and the right ventricle are measured Abbreviation: TTVR, transcatheter tricuspid valve replacement.

Transthoracic echocardiography is the initial imaging tool for grading TR severity, evaluating right ventricle (RV) size/function, and determining the underlying mechanism. TEE, especially with 3D imaging, provides a more detailed analysis of valvular anatomy including leaflet morphology, annular dilation, and subvalvular characteristics. TEE is also essential for identifying procedural hazards such as leaflet thickening, potential chordal entanglement, or pacemaker lead interference. In patients with torrential TR, special attention is needed to avoid underestimation of regurgitant severity, particularly when eccentric or multiple jets are present. An example of transthoracic echocardiography and TEE evaluation of a patient with torrential TR related to a carcinoid disease is provided in Figure 3.

#### Computed Tomography

Cardiac CT plays a central role in:

- Annular sizing and geometry (diameter, perimeter, and eccentricity)
- Preprocedural trajectory planning, particularly for aligning the transjugular delivery system with the annulus
- Visualizing adjacent structures, including the right coronary artery and conduction tissue pathways,
- Assessing right atrial and RV volumes, which may impact device alignment and anchoring stability.
- Measuring the angle between the interventricular septum and tricuspid annulus, too large or too small of the angle can cause challenges during leaflet grasping and increase the risk of lateral paravalvular leakage.

**Figure 4:**
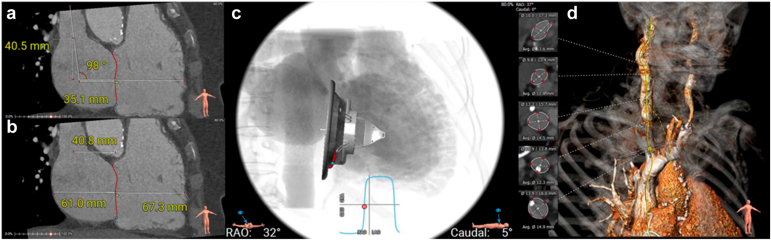
**Computed tomography analysis for TTVR screening using the Lux-Valve Plus system – part 2.** The angle between the superior vena cava and tricuspid annulus is measured **(a)**. The distances between the center of the tricuspid annulus and the free wall of the right ventricle and the right ventricle are measured **(b)**. The recommended projection angle (parallel tricuspid annulus plane) is calculated **(c)**. The access pathway is assessed including calcification, tortuosity and min average inner diameter of the vessel **(d)** Abbreviation: MPR, multiplanar reconstruction; TTVR, transcatheter tricuspid valve replacement.

**Figure 5:**
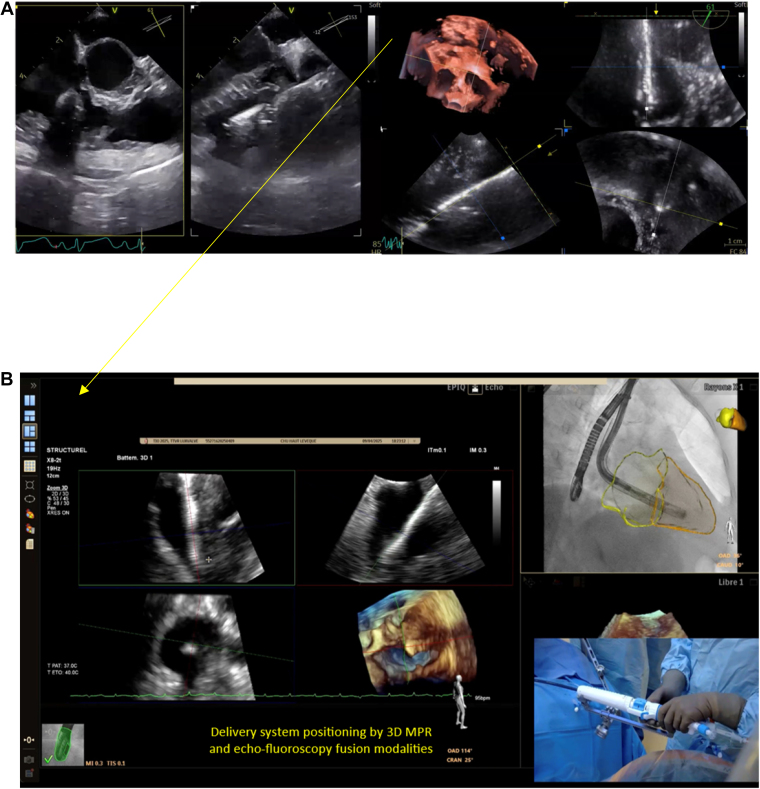
**Step-by-step intraprocedural guidance – part 1. (a)** The delivery system is steered into the RV without wire by gradually curving. **(b)** Coaxility and depth are checked (here using MPR). The base of the graspers are placed around 1 cm ventricular to the TV annulus. Abbreviations: RV, right ventricle; MPR, multiplanar reconstruction; TV, tricuspid valve.

In the case of LuX-Valve Plus, CT imaging also plays a crucial role in guiding septal anchor positioning by providing detailed reconstruction of the septal plane, which helps reduce the risk of conduction disturbances or improper deployment. An example of preprocedural planning with CT scan is provided in Figures 4 and 5.

Furthermore, the use of functional computed tomography angiography post-TTVR implant can also help understand device stability, leaflet mobility, and right-side chamber reverse remodeling. Those might be important secondary endpoints for future clinical trials in TTVR.

#### Considerations in Large Anatomies

The LuX-Valve Plus system expands the therapeutic window for patients with extreme annular dilation—a group frequently excluded from other TTVR systems. However, this anatomical setting brings specific procedural challenges.

- Loss of annular definition can impair precise imaging-based sizing.
- Massive right atrial enlargement often creates significant anatomical distortion of caval system angles and tricuspid annulus offset which may further complicate device coaxiality and navigation.
- Tethered or immobile leaflets, often seen in advanced cases, reduce effective leaflet capture zones.
- Ventricular septal wall remodeling by right heart dilatation requires meticulous planning for safe anchor deployment.

In such anatomical scenarios, preprocedural planning must balance device size selection with anchoring feasibility, aiming for stable engagement with minimal risk of paravalvular leak or malposition. Coordination between imaging specialists and interventionalists is critical to address this complexity.

### Step-by-step Intraprocedural Guidance

Successful LuX-Valve Plus implantation relies on meticulous intraprocedural coordination between interventionalists and imaging specialists. Given the anatomic complexity and size variability of the tricuspid valve apparatus, a structured, image-guided workflow is essential for procedural success and safety.

#### Vascular Access and Device Preparation

The LuX-Valve Plus is delivered via a transjugular venous approach, typically through the RIJ vein. A large bore sheath (33F) is introduced using percutaneous or surgical access under echocardiographic and fluoroscopic guidance. Systemic anticoagulation is initiated, and right heart pressures are measured to establish hemodynamic baseline values.

Maintaining a consistent volemic status between screening imaging and the procedural day is essential, as volume-related changes in right heart dimensions may directly influence valve sizing, anchoring stability, and procedural safety.

#### Crossing the Tricuspid Valve

A stiff guidewire is not anymore advanced across the tricuspid valve into the RV. It stays in the vena cava. The delivery system is then advanced over the wire into the right atrium and carefully positioned above the annulus without any stiff guidewire in the right ventricle.

#### Valve Positioning and Alignment

**Figure 6:**
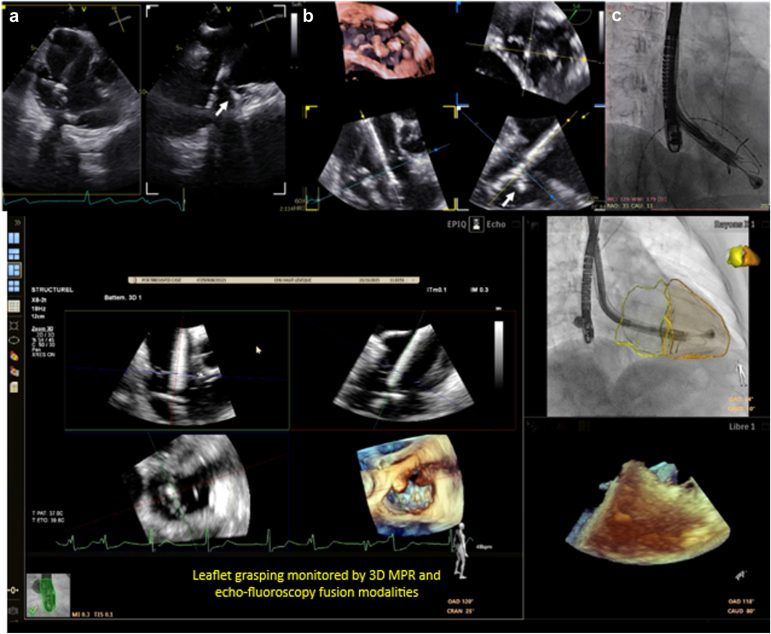
**Step-by-step intraprocedural guidance – part 2. (a-c)** Sheath is retrieved and the graspers exposed. The anterior and posterior graspers are deployed approximately 1 cm beneath the respective leaflets. Abbreviation: MPR, multiplanar reconstruction.

Precise coaxial alignment of the device with the tricuspid annular plane is achieved using biplane fluoroscopy and 3D TEE (Figure 6). The system allows for adjustment in depth, anteroposterior trajectory, and axial tilt to optimize leaflet capture and septal anchoring trajectory (the valve cannot be rotated before fully released). Once the annular plane is adequately centered, the anterior and posterior leaflet graspers are deployed.

#### Deployment and Anchoring

**Figure 7:**
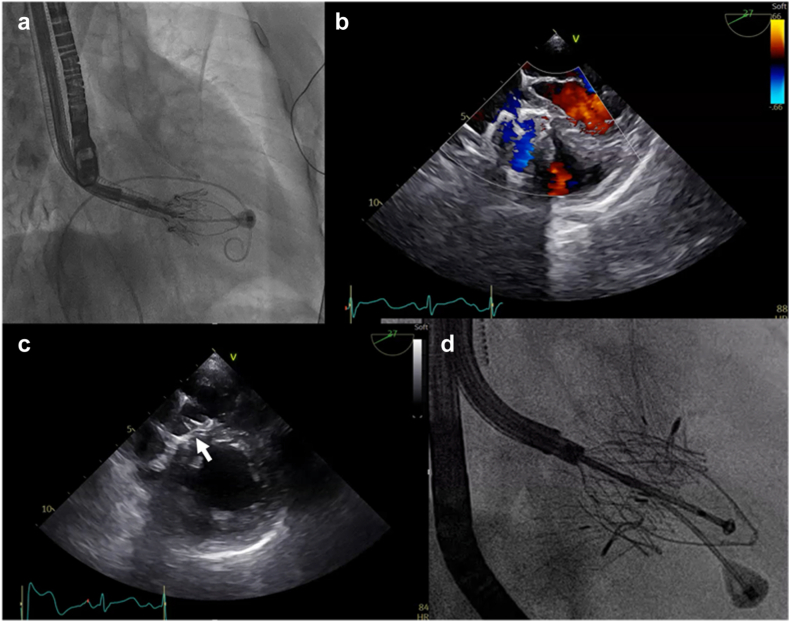
**Step-by-step intraprocedural guidance – part 3. (a and b)** Atrial skirt is released and final adjustments are made to minimze paravalvular leak. **(c and d)** The septal anchor is deployed.

**Figure 8:**
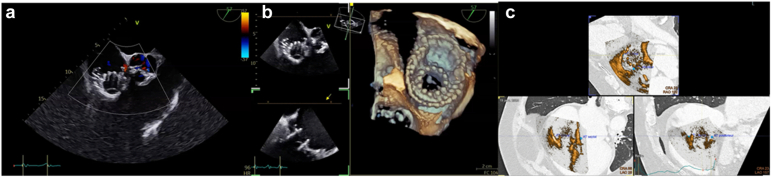
**Final Evaluation. (a)** Final device deployment and the degree of residual TR are assessed using Color Doppler imaging, **(b)** three-dimensional echocardiography with and without multiplanar reconstruction (MPR), and **(c)** echo-CT fusion imaging Abbreviations: TR, tricuspid regurgitation; CT, computed tomography.

After confirming appropriate leaflet engagement using 3D multiplanar reconstruction (MPR) modality by TEE (Figure 7), the ventricular portion of the valve is expanded, and the interventricular septal anchor is deployed into the position (Figure 8). A gentle septal pressure on the system ensures that the septal anchor is properly positioned by TEE (3D MPR, 4 chambers view 0°, or trans-gastric view). The device is gradually released, with continuous assessment of stability, central position, perpendicular trajectory (to the annulus) and regurgitation severity. Hemodynamic reassessment and color Doppler echocardiography confirm reduction of TR and assess for paravalvular leak or RV outflow tract obstruction. For this critical step in the procedure, a deep transgastric could be advised for. Intracardiac echocardiography imaging from the left ventricle for this step gives great certitude about septal anchor contact with the inter ventricular septum.

#### Final Checks and Hemostasis

The delivery system is withdrawn after confirming device stability. The access site is closed surgically or percutaneously, and postprocedural imaging is performed to assess valve function and RV performance (Figure 8). In early experience, postprocedural management after transjugular LuX-Valve Plus implantation has been straightforward, with no signal for conduction disturbances and no access-related complications, suggesting that the jugular approach may offer a particularly secure access route in elderly, comorbid patients compared with femoral access. Although currently available outcome data—summarized in the accompanying tables—are derived from early experience in expert centers and appear promising, the absence of pivotal randomized studies underscores the need for prospective trials to robustly assess the safety and efficacy of the LuX-Valve Plus system.

### Role of TEE, 3D Echo, and Fusion Imaging

TEE plays a central role in procedural guidance, especially when fluoroscopic annular markers are absent. Its key functions include (Figure 6, Figure 7, Figure 8): (1) performing the initial valve assessment by confirming the mechanism of disease, jet location, and leaflet mobility; (2) checking coaxiality during positioning (central position and perpendicular trajectory to the annulus), especially in noncircular annuli; (3) verifying leaflet capture, ensuring that both anterior and posterior graspers securely engage adequate leaflet tissue; (4) monitoring septal anchor deployment to prevent interaction with the conduction system or risk of septal perforation; and (5) evaluating outcomes postdeployment, including grading of residual TR and assessing right ventricular function.

3D TEE with MPR significantly enhances visualization by providing en face views of the tricuspid valve with the aortic valve at 11 o'clock, facilitating annular plane alignment and commissural orientation. This modality is critical for accurate spatial deployment, using anatomical landmarks such as the aortic valve and the septal-anterior commissure, and offers a surgical-like perspective to guide operator maneuvers.

Fusion imaging, when available, combines anatomical reconstructions with CT and real-time fluoroscopy and TEE. 4D-ICE could be considered as well. But this integration supports optimal septal anchor trajectory planning, verifies valve coaxiality during delivery system navigation, and helps identify structures that are poorly visualized under fluoroscopy alone (coronary sinus or valve plane).

With increasingly complex anatomies and high procedural stakes, LuX-Valve Plus implantation success relies not only on operator expertise but also on a well-coordinated imaging strategy. The seamless integration of advanced modalities such as 3D TEE and fusion imaging into real-time procedural decision-making is essential for optimal outcomes.

### Clinical and Procedural Outcomes

The international multicenter compassionate use registry reported by Stolz et al.^12^ represents the largest experience to date with the LuX-Valve Plus system.^13^ A total of 76 patients underwent transjugular TTVR between January 2022 and February 2024 across 15 international centers. The median patient age was 78 years, and nearly half were women. The vast majority presented with severe to torrential TR, and 75% received valve sizes ≥55 mm, reflecting treatment in patients with complex and dilated right heart anatomy.

#### Procedural Success and TR Reduction

Technical success was achieved in nearly all procedures, with TR reduced to ≤2+ in 94.7% of patients and to ≤1+ in 90.8% at the time of discharge. This favorable reduction was sustained at 30 days, with 95.0% of patients maintaining TR ≤ 2+ and 86.8% maintaining TR ≤ 1+. Importantly, all cases of residual TR were paravalvular in nature; no transvalvular regurgitation was observed.

#### Mortality and Major Clinical Events

In-hospital mortality occurred in 4 patients (5.3%), each presenting with significant pre-existing clinical risk. The 1-month survival rate was 94.4%. Notably, there was a significant improvement in New York Heart Association functional class at follow-up, indicating early symptomatic and clinical benefit. In the event of structural valve degeneration, reintervention strategies remain largely theoretical, with balloon-expandable valve-in-valve implantation appearing technically feasible based on bench testing and anatomical considerations, although clinical experience is currently lacking.

#### Complications

Major bleeding complications were observed in 6.6% of patients. The need for new pacemaker implantation was relatively low, occurring in 3.9% of the total cohort and in 5.7% of pacemaker-naïve individuals, despite the use of a septal anchoring mechanism. Surgical conversion during the index hospitalization was required in 5.3% of cases, underscoring the importance of comprehensive procedural planning. Paravalvular leak appears infrequent in early expert-center experience and is likely influenced by a learning-curve effect with progressive improvements in screening, sizing, and procedural technique; however, definitive assessment of paravalvular leak incidence and durability will require confirmation in prospective pivotal trials such as TRINITY. Although systematic postprocedural imaging surveillance—including venous Doppler assessment of the jugular access and echocardiographic monitoring of septal anchoring—is conceptually appropriate, early follow-up experience has been highly reassuring, with no clinically relevant access—or septal anchor–related complications reported to date.

And, although detailed adjudicated data on mortality, bleeding, and surgical conversion remain limited at this stage, early experience in expert centers appears encouraging, underscoring the importance of a careful and controlled assessment of postprocedural adverse events, as currently being undertaken in the prospective TRINITY pivotal trial.

#### Device Size Stratification

When stratified by device size (<55 mm vs. ≥55 mm), no significant differences were found in procedural success or complication rates. This finding is especially noteworthy, as it supports the safety and efficacy of the LuX-Valve Plus in patients with very large annuli—anatomic subsets typically excluded from other transcatheter treatment options.

## Discussion

### Interpretation of Early Data

The results from the LuX-Valve Plus registry demonstrate that transjugular TTVR is both feasible and effective, with high rates of TR elimination and clinical improvement, even in anatomies previously considered unsuitable for intervention. Despite the procedural complexity and the frailty of the treated population, early outcomes—including survival and New York Heart Association improvement—were favorable.

LuX-Valve Plus offers comparable TR reduction rates and acceptable safety, particularly in terms of pacemaker implantation. Currently EVOQUE is providing 44–56-mm size range valve sizes and a transfemoral delivery preferred.^8^ LuX-Valve Plus accommodates larger annuli and offering a transjugular route, potentially more suitable in distorted venous anatomies.^8^, ^9^, ^10^^,^^14^^,^^15^ When interpreted within a first-in-human and early-experience framework involving highly complex patients with advanced anatomical disease, the observed safety profile appears acceptable and is notably characterized by a low incidence of conduction disturbances compared with other TTVR platforms.

### Potential Patient Subgroups

These early findings are especially relevant for (1) patients with torrential TR and annular dimensions >55 mm, often excluded from other trials; (2) CIED carriers, where leaflet grasping is compromised, but anchoring and sealing with LuX-Valve remains feasible; and (3) patients contraindicated for TEER, due to wide coaptation gaps, leaflet tethering, or advanced right heart remodeling. By addressing anatomical and procedural limitations of TEER, LuX-Valve Plus emerges as a complementary solution within the TTVI spectrum, offering a potential first-line therapy in anatomically unfavorable cases.

### Future Perspectives

The field of TTVR is rapidly evolving, and the LuX-Valve Plus system has positioned itself as a promising platform for broader clinical investigation.

### Trials and Registries

Prospective multicenter studies are needed to validate the early compassionate use findings and define optimal patient selection criteria. Randomized comparisons with TEER (ineligible vs. marginal anatomies) and surgical options are essential to guide treatment algorithms. Dedicated imaging registries could help refine procedural protocols, particularly for anchor deployment and device sizing.

### Durability and Long-Term Surveillance

Long-term valve performance, durability, and structural valve deterioration remain unknown and will be critical to establish. Moreover, evaluation of right ventricular reverse remodeling post-TTVR may offer prognostic insight and justify earlier intervention. Finally, standardized postprocedure anticoagulation strategies must be defined, especially given the risk of thrombosis in low-pressure, low-flow RV environments.

Ultimately, widespread adoption of LuX-Valve Plus will depend on integrating procedural training, real-world data, and imaging standardization into a cohesive clinical framework.

## Review Statement

The review of this manuscript was managed by Guest Editor: Firas Zahr, MD.

## Disclosure Statement

The authors report a pending patent. The authors report no conflict of interest.
